# Uncovering New Insights and Misconceptions on the Effectiveness of Phosphate Solubilizing Rhizobacteria in Plants: A Meta-Analysis

**DOI:** 10.3389/fpls.2022.858804

**Published:** 2022-03-02

**Authors:** Noémie De Zutter, Maarten Ameye, Boris Bekaert, Jan Verwaeren, Leen De Gelder, Kris Audenaert

**Affiliations:** ^1^Laboratory of Applied Mycology and Phenomics (LAMP), Department of Plants and Crops, Faculty of Bioscience Engineering, Ghent University, Ghent, Belgium; ^2^Laboratory of Environmental Biotechnology, Department of Biotechnology, Faculty of Bioscience Engineering, Ghent University, Ghent, Belgium; ^3^Research Unit Knowledge-based Systems (KERMIT), Department of Data Analysis and Mathematical Modelling, Ghent University, Ghent, Belgium

**Keywords:** phosphorus deficiency, meta-analysis, plant-bacteria interactions, plant nutrition, phosphate solubilizing bacteria

## Abstract

As the awareness on the ecological impact of chemical phosphate fertilizers grows, research turns to sustainable alternatives such as the implementation of phosphate solubilizing bacteria (PSB), which make largely immobile phosphorous reserves in soils available for uptake by plants. In this review, we introduce the mechanisms by which plants facilitate P-uptake and illustrate how PSB improve the bioavailability of this nutrient. Next, the effectiveness of PSB on increasing plant biomass and P-uptake is assessed using a meta-analysis approach. Our review demonstrates that improved P-uptake does not always translate in improved plant height and biomass. We show that the effect of PSB on plants does not provide an added benefit when using bacterial consortia compared to single strains. Moreover, the commonly reported species for P-solubilization, *Bacillus* spp. and *Pseudomonas* spp., are outperformed by the scarcely implemented *Burkholderia* spp. Despite the similar responses to PSB in monocots and eudicots, species responsiveness to PSB varies within both clades. Remarkably, the meta-analysis challenges the common belief that PSB are less effective under field conditions compared to greenhouse conditions. This review provides innovative insights and identifies key questions for future research on PSB to promote their implementation in agriculture.

## Introduction

Phosphorus (P) is vital for plant growth and development as it is an essential component in biomolecules such as nucleic acids, phospholipids, and ATP (Schachtman et al., 1998; Tian et al., 2021). For P-uptake, adult plants depend entirely on their root system to retrieve the available P from the soil as orthophosphates (Shen et al., 2011). However, due to their highly reactive nature, orthophosphates are prone to adsorption onto mineral surfaces (e.g., clay minerals), precipitation into various salts (e.g., Ca-, Fe-, Al-, and Mn-phosphates) or immobilization into organic phosphorus (Owen et al., 2015). Additionally, the processes making P inaccessible for plants are influenced by abiotic factors such as soil pH, soil texture and aeration, soil temperature, and soil composition (Amery and Vandecasteele, 2015).

### Plant Responses to P-Deficiency

**FIGURE 1:**
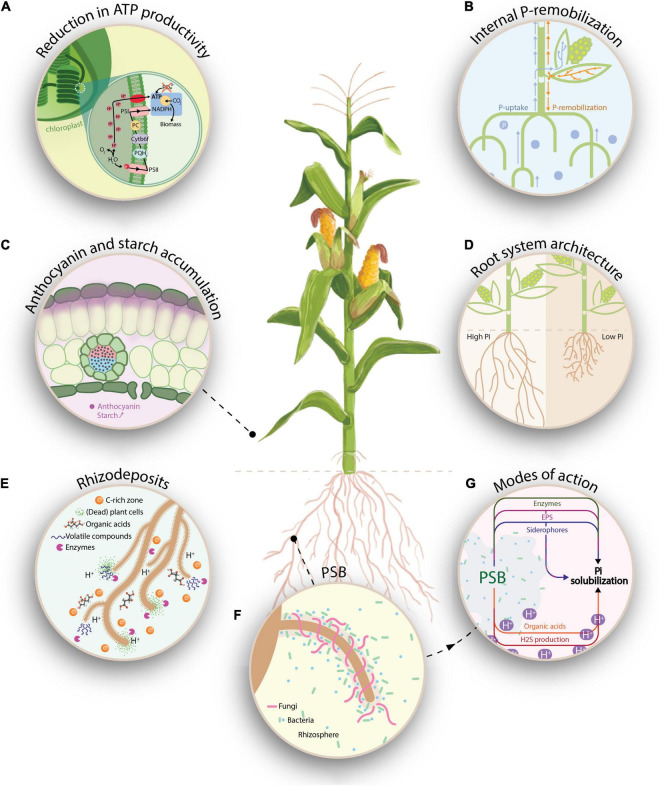
Graphical representation on how P-deficiency affects plant physiology and morphology **(A–D)**, how it affects the rhizobiome **(E,F)** and how PSB can interfere with the bioavailable P-fraction **(G)**. **(A)** Low levels of P lead to reduced ATP synthesis, causing the accumulation of protons in the thylakoid, which results in a disruption of the linear electron flow. In the Calvin cycle, these ATP-limitations reduce the net CO_2_ assimilation and cause NADP^+^ to remain in its reduced form. **(B)** Plants can absorb P through the soil solution, which is subsequently transported through the xylem. Upon P-depletion, internal P-remobilization occurs, whereas P is translocated through the phloem from mature leaves to younger tissue. **(C)** In P-deprived plants, enhanced starch accumulation occurs in the chloroplasts as a result of redirecting triosephosphates to the starch biosynthesis in order to release P. In the epidermal layer, anthocyanins are formed through the flavonoid pathway and serve as a photo-protectants against photo-oxidative stress. **(D)** Adaptations in the plant root system under P-deficiency (e.g., reduced primary root growth and increased lateral root formation) allow plants to access a larger volume of the soil solution. **(E)** The release of rhizodeposits, which comprises of (dead) plant cells, border cells, root exudates (e.g., organic acids and enzymes) and volatile compounds causes the rhizosphere to be a nutrient (carbon) rich zone. Upon P-deficiency, specific root exudates such as GABA (gamma-aminobutyric acid) and carbohydrates are released, serving as chemo-attractants for (beneficial) PSB. **(F)** Recruitment of PSB and AMF in the rhizosphere to enhance bioavailable P, hence improving the plant’s nutritional status. **(G)** Representation of potential modes of action (MOA) by which PSB can render P available for the plant. Amongst others, the production of organic acids and H2S cause an acidification of the rhizosphere environment, rendering inorganic P bioavailable. Additionally, inorganic P-solubilization can also by mediated by the release of siderophores and exopolysaccharides (EPS), which bind to metal ions (Fe^3+^ resp. Al^3+^, Ca^2+^, and Fe^3+^). Finally, organic P-mineralization can be regulated by three groups of enzymes, namely, non-specific phosphatases, phytases and C-P-lyases. Inspired by de Bang et al. (2021).

As sedentary organisms, plants have developed sophisticated ways to maintain their P homeostasis when P is scarcely available in soils (Rouached et al., 2010). Several physiological responses have been reported upon P-deprivation. For example, in the light reactions of photosynthesis, low levels of phosphate lead to reduced ATP synthesis (Figure 1A). In the Calvin cycle, these ATP-limitations cause a reduced net CO_2_ assimilation and NADP^+^ to remain in its reduced form (NADPH) (de Bang et al., 2021). Upon P-depletion, the accumulation of phosphorylated intermediates is bypassed by channeling triosephosphate molecules to starch biosynthesis and to the accumulation of non-phosphorylated products, hence releasing phosphate (Hernández and Munné-Bosch, 2015). Additionally, other adaptive responses comprise internal P-remobilization from source leaves to sinks within the plant (Figure 1B). At a subcellular level, P-remobilization occurs between storage pools in vacuoles and other organelles such as chloroplasts. At the level of membrane lipids, physiological changes take place and phospholipids are replaced by galactolipids (Härtel et al., 2000; Wang et al., 2021). A last typical metabolic hallmark of P-deficiency, especially in C4 plants, is the accumulation of anthocyanins. Anthocyanins are formed in epidermal cell layers through the flavonoid metabolism upon P-deficiency and act as protectant to alleviate the photooxidative stress (Figure 1C; Jiang et al., 2007; Hernández and Munné-Bosch, 2015).

Besides the internal adaptation of the plant’s physiology and metabolism, plants invest in the external acquisition of P from soils by optimizing their rhizosphere environment. At a macromorphological scale, modifications of the root architecture in response to low P influence the P acquisition. These adaptations include reduced primary root growth, increased lateral root development, increased development of root hairs, and formation of cluster roots (Niu et al., 2013), in which plant hormones such as auxins, ethylene, abscisic acid, and cytokinins play an important role. An increased root-to-shoot ratio allows plants to access larger volumes of the soil solution, consequently improving P-uptake (Figure 1D; Lynch and Brown, 2008). Additionally, at a biochemical level, the excretion of root exudates can influence the P-availability in the rhizosphere. For example, increased extracellular enzymes activity, such as phytase and acid phosphatase activity, can release P from organic substrates (Dakora and Phillips, 2002). Finally, the excretion of organic acid anions as a response to P-deficiency induces acidification in the rhizosphere, solubilizing (calcium-)P and rendering it available to plants (Hinsinger, 2001).

### Rhizodeposits and Microbial Crosstalk Shape the Rhizosphere Environment

Rhizodeposits comprise (dead) plant cells, border cells, mucilage, root exudates, and volatile compounds (Tian et al., 2020), however, the proportion and amounts are dependent on plant species, variety, age, lateral position on the root, and (a)biotic conditions (Sasse et al., 2018). Rhizodeposits do not only improve P-availability, e.g., through pH-modifications or enzyme secretion, but also serve as signaling molecules that allow the plant to actively recruit bacteria and fungi from the bulk soil to the rhizosphere. The rhizosphere is a complex, carbon rich zone on the interface between the plant root and bulk soil, in which the release of rhizodeposits influences the diversity, composition, and activity of the rhizobiome (Figure 1E; Sasse et al., 2018; Liu et al., 2021). Anticipating (a)biotic stresses, plants actively shape their rhizobiome by exuding chemo-attractants, enhancing the root colonizing ability of certain microorganisms (Berendsen et al., 2012; Feng et al., 2019). Specifically for plants grown under P-deficiency, the release of gamma-aminobutyric acid (GABA) and carbohydrates through root exudates is stimulated. GABA has been associated with signaling in several abiotic stress responses and may act as a signaling compound in P-deprived plants (Kinnersley and Turano, 2000; Bouche and Fromm, 2004), while carbohydrates induce bacterial genes involved in chemotaxis and motility (Carvalhais et al., 2013). Once the microorganisms have reached the rhizosphere, the rhizobiome composition is not only shaped by the plant, but is influenced by mutual interactions between the members of the microbial community (Kai et al., 2016; Sasse et al., 2018).

After recruiting microorganisms to counter P-deficiency, the plant depends on those microorganisms to alleviate P-starvation (Figure 1F). Phosphate solubilizing bacteria (PSB) and arbuscular mycorrhizal fungi (AMF) are types of, respectively, plant growth promoting rhizobacteria (PGPR) and fungi (PGPF) which are able to solubilize P through different mechanisms, rendering P available for plants. AMF engage in a mutualistic, symbiotic partnership with plants, where the heterotrophic fungi depend on the plant for organic carbon sources, while the plant depends on the fungi for its P-supply. AMF enhance plant P-uptake primarily by expanding the mycorrhizal hyphal network, serving as an extension of the root system, to reach beyond the rhizosphere (Begum et al., 2019; Etesami et al., 2021). Despite not being included in present meta-analysis, the dual role of AMF in P-solubilization is of paramount importance: besides solubilizing P, they might interact with PSB when co-occurring in and on roots. For example, AMF provide a niche for bacteria through their extraradical hyphal network (Zhang et al., 2016). Depending on the P-availability in soils, AMF might supply the required carbon through hyphal exudates to PSB, while PSB in its turn might help AMF by supplying P (Etesami et al., 2021). However, under low P-availability, PSB and AMF might compete with one another for this nutrient (Zhang et al., 2016). Comparable to plant roots, AMF hyphae produce exudates, which in turn might alter the soil microbial community composition (Scheublin et al., 2010). For example, Nuccio et al. (2013) described that the relative abundance of *Firmicutes* in soil was positively influenced by AMF, while the relative abundance of *Actinobacteria* was negatively influenced.

### Phosphate Solubilizing Bacteria: Modes of Action

Phosphate solubilizing bacteria can release P from both inorganic and organic sources through solubilization and biochemical- and biological mineralization, respectively (Sharma et al., 2013). The modes-of-action by which microorganisms solubilize P are well documented in literature and will be briefly summarized (Figure 1G; Zaidi et al., 2009; Sharma et al., 2013; Alori et al., 2017; Rawat et al., 2021). Inorganic P-solubilization is mediated by organic acid production, siderophore production, H_2_S-production, and metal-binding exopolysaccharides (EPS). Biochemical mineralization of organic P is regulated by three enzyme groups: non-specific phosphatases, phytases, and C-P lyases. Finally, biological mineralization of organic P comprises the release of P during substrate degradation. By rapidly immobilizing bioavailable P, PSB may serve as P-sinks, whereas upon P-release from their cells, they become a source of P to plants (Sharma et al., 2013; Raymond et al., 2021).

Apart from their inherent phosphate solubilizing mechanisms, microorganisms can also mitigate P-deficiency by influencing the plant’s metabolism and hormonal pathways, for example, through the production of, or interference with phytohormones (Friesen et al., 2011; Lapsansky et al., 2016). A well-known example hereof is the bacterial production of the auxin indole-acetic acid (IAA), which provokes changes in the plant root phenotype (Nacry et al., 2005). The combination of plant- and bacterial IAA activates the auxin response factors and subsequently regulates the P-response (Friesen et al., 2011; Lim and Kim, 2013).

Summarizing, the use of PSB can both directly and indirectly influence plant health, and its nutritional status. Taking the above into account, the application of PSB in agricultural systems provides a sustainable alternative for, or a complement to, chemical P-fertilizers and has spurred many research groups to explore highly promising PSB.

### Phosphate Solubilizing Bacteria: From Isolation and Selection to Application

In search for these highly promising PSB strains, the most adopted isolation and selection pipeline is a top-down strategy which comprises large scale *in vitro* screenings on selective growth media, followed by preliminary *in planta* screenings of the best performing isolates under greenhouse conditions, which finally results in small-scaled field experiments with the “top-of-the-class” isolates. There are several factors affecting the success and failure of PSB in the field, amongst which their rhizosphere competence is a critical one.

Numerous research articles have been published describing the (variable) performance of PSB in association with a host plant. Consensus on the (in)effectiveness of PSB is lacking, and a recent article critically reviews the shortcomings of PSB (Raymond et al., 2021). However, insights in the reasons behind this potential (in)effectiveness constitutes a knowledge gap in literature, demonstrating the necessity of a comprehensive literature review by means of a meta-analysis. In this study, we evaluated the effect of several variables of PSB’s effectiveness on crop biomass growth (root and shoot) and P-uptake. Variation caused by the following parameters was evaluated: (1) at plant level: plant group and plant species; (2) at inoculum level: inoculum composition, application, and species distribution; and (3) at experiment level: experiment type, fertilizer treatment, and soil acidity.

## Methods

### Search Strategy and Data Collection

**FIGURE 2:**
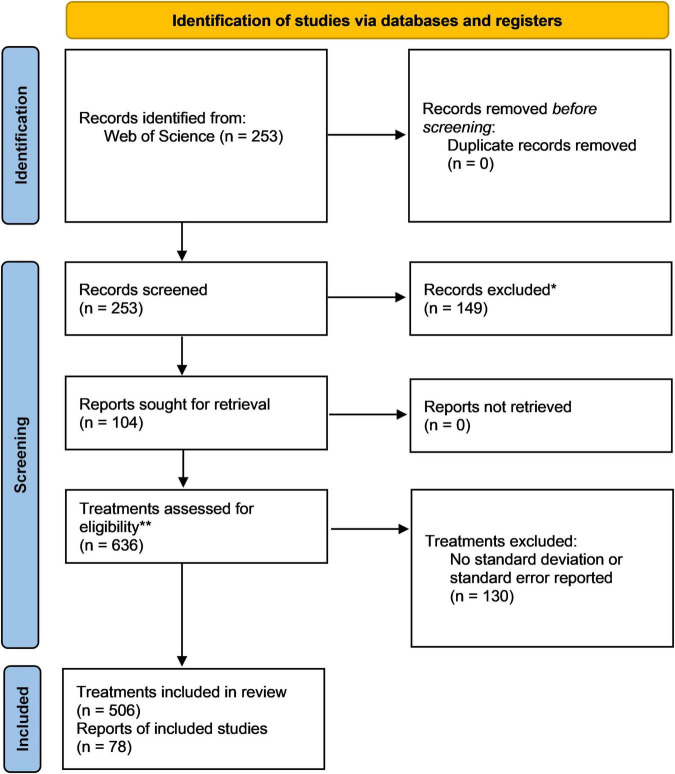
PRISMA flow chart demonstrating the data acquisition, selection and processing pipeline used in this meta-analysis (Page et al., 2021).

A thorough literature research was conducted in Web Of Science using the keywords “phosphate solubilizing bacteria” (*title*) AND “plant growth” (*topic*), resulting in 253 identified records published between April 1976 and May 2021. After filtering and extracting information from relevant studies, 104 articles were retained (Figure 2 and Supplementary Note 1). The meta-analysis was conducted on 506 single treatments to evaluate the effect of PSB-inoculation on plant P-uptake and shoot- and root biomass. The effect size of each treatment was calculated by means of the Hedges’ g (unless indicated otherwise), which represents the standardized difference of the means between the treatment and control (Hedges, 1981). A Hedges’ g of zero indicates no treatment effect is present, while a Hedges’ g of 1 indicates that two groups differ by 1 standard deviation.

After removing extreme values (as defined by Tukey, 1977), data robustness and publication bias were evaluated by means of the fail-safe number (FSN) and visual inspection of funnel plots using the R-packages “*meta*” and “*metafor*” (Viechtbauer, 2010; Balduzzi et al., 2019). Different factors that might introduce variation in plant response to PSB were evaluated and the data were statistically compared at the 5% significance level by means of ANOVA analysis, followed by *post-hoc* Dunnett T3 tests.

### Fail-Safe Number to Evaluate Data Robustness

To verify the robustness of the observed treatment effects, the fail-safe number was calculated as reported by Rosenberg (2005). This number represents the amount of observations displaying a non-significant treatment effect that should be added to the meta-analysis to disprove the observed effects. The fail-safe number for each effect (P-uptake and shoot- and root biomass) was calculated in RStudio V4.0.2 using the package “*meta*” (Table 1; Balduzzi et al., 2019).

**TABLE 1:** Overview of the fail-safe number per effect type (P-uptake, shoot biomass, and root biomass) as determined by the Rosenberg’s approach and the respective number of studies included in this meta-analysis.

Treatment	Fail-safe number	Number of studies
P-uptake	69,305	327
Shoot biomass	92,433	378
Root biomass	60,940	283

### Funnel Plots to Evaluate Publication Bias

Funnel plots were generated in RStudio V4.0.2 using the package “*meta*” in order to verify the presence of potential publication bias (i.e., whether the conclusion of a study influences the decision to publish it; Sterne and Egger, 2001; Balduzzi et al., 2019). Duval and Tweedie’s (2000) trim and fill method was used to help interpret the results. In the absence of publication bias, a symmetrical distribution of the studies around the mean treatment effect will be observed when both positive and negative effects compared to the control are expected. The funnel plots were asymmetrical for each effect (Supplementary Figure 1), however, in our opinion, this is by and large attributed to a lack of negative effect sizes when working with biostimulant traits. After trimming and filling the data, a rather small decrease in mean treatment effect is observed (Supplementary Figure 2). Combined with the high fail-safe number, we can state that the data are robust.

## Results

### Plant Growth Promotion Is Not Always Related to Improved P-Uptake

**FIGURE 3:**
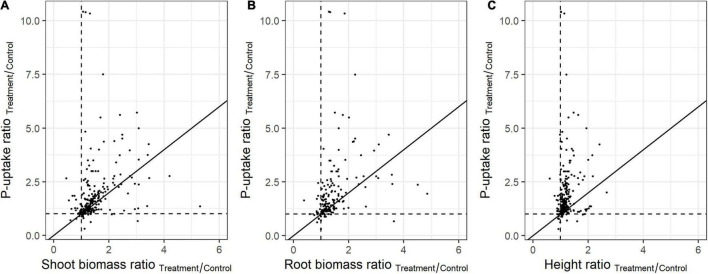
Relationship between plant P-uptake and **(A)** shoot biomass (*n* = 261), **(B)** root biomass (*n* = 190), and **(C)** length (*n* = 252) upon bacterial inoculation. Values represent the ratio of the means (P-uptake, biomass, and length) between the treatment and control. Black lines represents the first bisector (y = x).

In order to assess the *in planta* effect of highly promising PSB, plant height or biomass are often monitored. The representativeness of these parameters for the plant’s P-uptake was evaluated in the present meta-analysis. To this end, the ratio of the means (P-uptake, biomass, and height) between the treatment and control plants were used (independent on the experiment size and variation). When comparing the effect of PSB on plant P-uptake versus biomass and height at full growth, many datapoints are distant from the first bisector, which refers to differences between the P-uptake and the effect on the plant’s biomass or height (Figure 3). The PSB which caused the highest impact on shoot-biomass and height, showed little to no effect on plant P-uptake, and vice versa.

Additionally, the predictive power of the plant biomass and height is dependent on the crop type (Supplementary Figure 3). For example, in monocots, maize biomass is aberrant from the first bisector, while rice biomass is adversely affected, showing a reciprocal relationship with plant P-uptake (Supplementary Figures 3A–C). In eudicots, *Camellia oleifera* and Mung bean biomass are aberrant to the first bisector, while all other eudicots follow the first bisector (Supplementary Figures 3D–F). The predictive power of plant height proved to be limited in both monocots and eudicots (Supplementary Figures 3C,F).

### The Application of Phosphate Solubilizing Bacteria Has Different Effects Within the Plant Families

**FIGURE 4:**
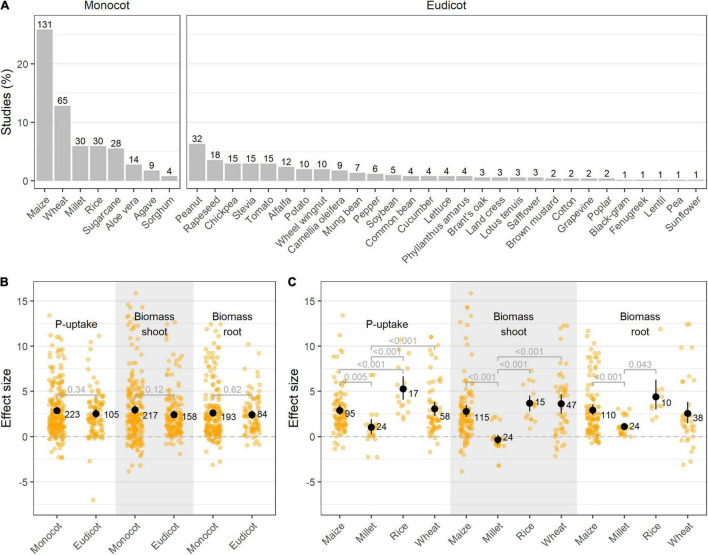
**(A)** Visualization of the different crops included in this meta-analysis per group (monocotyledon vs. dicotyledon plants). Bars represent the relative frequency of the studies as opposed to the complete dataset (% studies), while absolute numbers represent the amount of observations; **(B)** Effect size on P-uptake, shoot- and root biomass upon PSB inoculation in monocot vs. dicot plants; and **(C)** Effect size on P-uptake, shoot- and root biomass upon PSB inoculation in the four most abundant monocot crops reported in this meta-analysis. Values represent the means ± 95% c.i. Statistical differences were calculated by means of ANOVA and *post-hoc* Dunnett T3 tests.

Although P-deficiency in plants is a widespread problem, research on the use of PSB as an ecological alternative or complement to chemical fertilizers is mainly situated in (Southern-) America and Asia (Supplementary Figure 4). In these studies, monocotyledons are more often used as test plants than dicotyledons (311 resp. 195 studies), with maize (*Zea mays* L.) being prominently used in 25.9% of all studies (Figure 4A). As cereal crops account for the largest total cultivation and production area worldwide, with maize (*Zea mays* L.), rice (*Oryza sativa* L.), wheat (*Triticum aestivum* L.), and sugarcane (*Saccharum officinarum* L.) as leading crops (FAOSTAT, 2021), it is expected that they represent the majority amongst the test subjects.

The application of PSB to both monocotyledon (monocots) and dicotyledon plants (eudicots) resulted in an overall positive effect on plant P-uptake, root- and shoot biomass, albeit no significant difference was found between PSB’s effectiveness on monocots and eudicots (Figure 4B). In addition, no differences in plant response between bacterial genera used as PSB were uncovered when comparing monocots and eudicots (data not shown). Moreover, in 92.7% of all studies, the bacterial species tested on the monocot (resp. eudicot) was also tested on an eudicot (resp. monocot) in another study. When focusing on the *Poaceae*, a clear differentiation between the plant species can be observed, with rice showing a tendency for superior P-uptake upon PSB inoculation (Figure 4C).

### Multispecies Inocula Do Not Always Provide Added Value Over Single-Species Inocula

**FIGURE 5:**
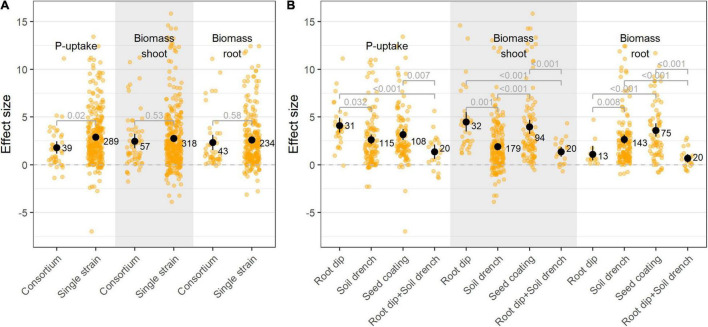
**(A)** Effect size on P-uptake, shoot- and root biomass upon single and multiple species inoculation; and **(B)** Effect size on P-uptake, shoot- and root biomass upon bacterial inoculation by means of root dip, soil drench, seed coating, and combined root dip and soil drench. Values (black dots) represent the means ± 95% c.i. Orange jitter represent the distribution of all datapoints. Statistical differences were calculated by means of ANOVA and *post-hoc* Dunnett T3 tests.

The primary selection of PSB is commonly done through *in vitro* screenings, in which bacteria are tested for their P-solubilizing capacity on selective growth media. Once potential PSB’s are singled out, they can be applied to a host plant, either as a single-species inoculum or in combination with other (bacterial) isolates as a multispecies inoculum. Plant parameters are improved upon both single and multispecies inoculation, albeit P-uptake proved to be superior upon single species inoculation (Figure 5A). We identified 13 studies that tested both single isolates and combinations of those specific isolates in the same paper, or in a back-to-back paper. These records were selected, resulting in 87 single treatments (64 single-species inocula, 23 multispecies inocula). When comparing those specific cases, no significant differences in plant P-uptake were identified between single and multispecies inoculation (*p* = 0.85; Supplementary Figure 5).

### Phosphate Solubilizing Bacteria’s Effectiveness Is Influenced by the Application Method

The most adopted bacterial inoculation strategies were soil drench (41.7% of all studies), followed by seed coating (28.1%) and root dip (11.5%). Other inoculation methods (e.g., spray inoculation) accounted for less than 5% of all studies and were omitted from this particular analysis. Bacterial inoculation by means of root dip resulted in the largest increase in P-uptake and shoot biomass, followed by seed coating (Figure 5B). The combined application of bacteria by means of root dip and soil drench resulted in a lower P-effect compared to their individual applications.

### *Burkholderia* spp. Outperform *Bacillus* spp. and *Pseudomonas* spp. for Improved P-Uptake

**FIGURE 6:**
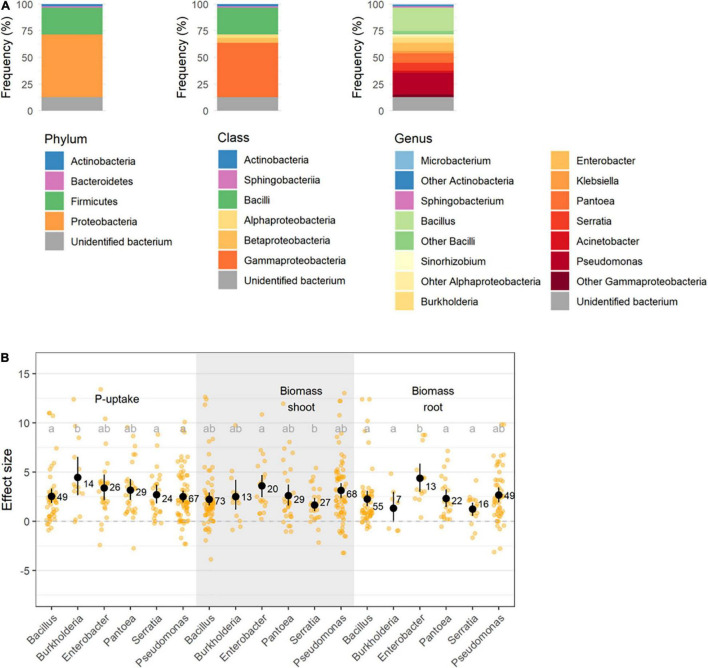
**(A)** Representation of bacterial phyla, classes, and genera used in this meta-analysis; and **(B)** Effect size on P-uptake, shoot- and root biomass upon inoculation with the six most abundant bacterial genera. Values represent the means ± 95% c.i. Statistical differences were calculated by means of ANOVA and *post-hoc* Dunnett T3 tests.

Focusing on the studies using single species inocula, *Bacillus* spp. and *Pseudomonas* spp. have primarily been tested for their phosphate solubilizing capabilities (21.7% resp. 20.3%, Figure 6A). A pairwise comparison between the six most abundant bacterial species (*n* > 10 studies) shows that application of the scarcely implemented *Burkholderia* spp. outperforms the more commonly reported *Bacillus* spp. and *Pseudomonas* spp. for increased plant P-uptake, while the application of *Enterobacter* spp. resulted in the highest increased shoot- and root biomass (Figure 6B). Although the formulation and storage of *Bacillus* spp. is straightforward due to their sporulation abilities, their beneficial effect is considered ambiguous due to their poor root colonizing capacity (Gao et al., 2016). However, in this meta-analysis the beneficial effect of *Bacillus* spp. was confirmed, displaying positive effects on plant P-uptake, shoot- and root biomass in resp. 92, 88, and 89% of all studies using *Bacillus* spp*.*

### Phosphate Solubilizing Bacteria Are Effective Under Field Conditions

**FIGURE 7:**
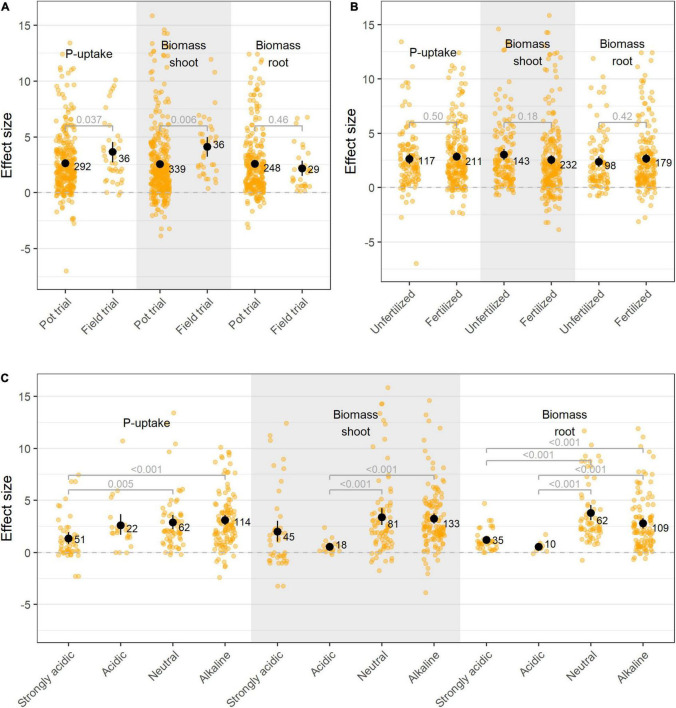
**(A)** Effect size on P-uptake, shoot- and root biomass upon bacterial inoculation applied in pot- and field trials; **(B)** Effect size on P-uptake, shoot- and root biomass upon bacterial inoculation on plants grown in fertilized versus unfertilized soil; and **(C)** Effect size on P-uptake, shoot- and root biomass upon bacterial inoculation on plants under different soil acidities. Values represent the means ± 95% c.i. Statistical differences were calculated by means of ANOVA and *post-hoc* Dunnett T3 tests.

The majority of the studies in this meta-analysis were conducted in pot trials under greenhouse conditions (90% of all studies). When comparing pot and field trials, our analysis of the data does not support the generally accepted notion that PSB are less effective when tested in the field (Figure 7A). To investigate this, a subset of papers were selected in which the same isolate(s) were tested in both pot- and field trials. Application of these isolates resulted in similar effect sizes in the field trials as in their respective pot trials (Supplementary Figure 6). However, care should be taken when interpreting the increased performance on the field versus in pots (Figure 7A), as a bias toward isolates which perform well in pot trials are selected for field trials. Additionally, the effectiveness of bacterial isolates in greenhouse or field experiments was not influenced by the application of phosphate fertilizer (Figure 7B).

### Soil pH Proves to Be an Important Factor for Phosphate Solubilizing Bacteria’s Effectiveness

P-availability and P-type are influenced by soil pH. In acidic soils, the majority of P is precipitated into iron- and aluminum phosphates, while in alkaline soils P is primary fixed by calcium (Penn and Camberato, 2019). In present meta-analysis, research was mainly situated in low pH-zones (Supplementary Figures 4, 7), however, the majority of the trials were conducted under neutral to alkaline conditions (75% of all studies in which pH was indicated; *n*_all_ = 369). Additionally, when reported, the majority of the preliminary *in vitro* screenings were also conducted in neutral to alkaline medium, supplemented with tri-calcium phosphate (TCP, 65%). PSB’s effectiveness proved to be dependent on the pH of the soil, with bacteria introduced in alkaline soils showing better treatment effects (Figure 7C). This effect proved to be independent on the crop type, bacterial species or P-fertilizer, as within resp. specific crop types, bacterial species and P-fertilizer groups, the same effects were observed.

## Discussion

### Plant Growth Promotion Is Not Always Related to Improved P-Uptake

The relevance of plant height, shoot and root biomass as proxies for plant P-uptake was evaluated for all studies included in this meta-analysis. The use of these criteria should be handled with care, since their predictive power dependent on the crop type. Plant relative height proved to be an ineffective parameter for the assessment of a crop’s P-status, both in monocots and eudicots. Shoot biomass proved to be a good proxy for most of the eudicots, but not for monocots such as maize and rice. This might be the result of so called “excess-uptake” or “luxury uptake,” which means that plant P-uptake reached beyond the essential uptake necessary for immediate growth (Ågren, 2008; Sims et al., 2012; Gagnon et al., 2020). This is an important observation to take into account when selecting high potential PSB for pot or field trials, since biomass is recurrently chosen as the indicative parameter to evaluate a plant’s P-status (36.8% of all studies in present meta-analysis). Recently, research has also turned to the non-destructive estimation of plant health and plant nutrient status. The use of multi- and hyperspectral imaging for the monitoring and estimation of a crop’s P-status is an uprising technique and could be used as a tool to better non-destructively assess the claim of P solubilization instead of plant growth promotion (Gitelson et al., 2009; Rouphael et al., 2018; De Zutter et al., 2021).

### Differential Responses Between Plant Families and Their Growth Conditions Complicate the Use of a Generalistic Inoculum

It is well described in literature that the P-status of plants is tightly regulated through plant hormone crosstalk, and although sharing several branches, these plant hormone regulatory networks differ considerably in monocots and eudicots (Ha and Tran, 2014; Nelissen et al., 2016; Chen et al., 2018). Despite the regulatory differences regarding root development, the response to exogenously applied PSB is not significantly different between both plant clades.

The apparently well conserved trait of plants to react to PSB activity merges with the holobiont theorem, which states that plants as organisms from an evolutionary perspective have always been dependent on their interaction with microorganisms (Lyu et al., 2021a). A major constraint in the transition from aquatic to terrestrial plant life, was the inadequate water and nutrient supply. To meet these requirements and facilitate nutrient acquisition, plants engaged in symbiotic relationships with soil microorganisms such as PSB (Lyu et al., 2021b). The co-evolution between a plant and its microbiome has led to a highly structured rhizosphere characterized by an interactive rhizobiome, in which cross-kingdom communication results in improved performance for both partners (Wallenstein, 2017).

Within the monocots, rice plants experienced the largest increase in plant P-uptake upon bacterial inoculation. A possible explanation for this phenomenon lies within the cultivation method of rice. Rice is often grown in flooded wetlands called paddy soils, which provides a feasible environment for both aerobic and (facultative) anaerobic bacteria (Suzuki, 1967). Additionally, in these soils, P is generally adsorbed onto iron- and aluminum minerals or precipitated into iron- and aluminum phosphates (Yan et al., 2017). Under anaerobic conditions, bacteria capable of performing sulfur reduction (SO_4_^2–^ to H_2_S) might contribute to the release of P from iron phosphate present in the paddy soils (Sharma et al., 2013). When selecting PSB for plant growth promoting trials, not only the bacteria and host plant, but also the intended environment should therefore be taken into account.

### The Use of a Multi-Species Inoculum Should Be Well Considered and Designed

Multi-species microbial consortia are being increasingly used in agriculture with the aim of plant growth promotion. They are composed of a bacterial mixture, in which each bacterium might consist of a different mechanism to promote plant growth and health (Santos et al., 2019). However, in this meta-analysis, the effect of multi-species inoculation on plant P-uptake, shoot- and root biomass was not higher than that of single species inoculation. An important consideration is that the research included in this meta-analysis combined only two to four bacterial taxa, in which the rationale to pool these strains was limited to their individual positive effect. Notwithstanding the efforts of research to develop multispecies inocula, it is essential to recognize the possible trade-offs (e.g., competition for nutrients) within these consortia. Therefore, the combination of several microorganisms should be designed with care.

Recent studies concerning synthetic microbial communities (SynComs) aim to expand the current application strategies of single and multispecies inocula (de Souza et al., 2020; Marín et al., 2021). SynComs are composed of PGPR species and strengthened with both accessory and keystone species to obtain a robust diversity and maintain the functional stability, respectively (Sánchez-Cañizares et al., 2017). These synthetic communities must form associations with the rest of the microbiome to mimic the interactive rhizobiome and function in the plant holobiont. We suggest that further research concerning PSB should explore the formulation of these types of SynComs anticipate competitive exclusion in the rhizosphere (Pandey et al., 2012; Rubin et al., 2017) and to establish long-term stable survival of the SynCom members in the soil.

Bearing in mind the added values of SynComs, the combined application of AMF and PSB should also be explored. It is known that AMF and (phosphate solubilizing) bacteria can engage in synergistic interactions to improve the plant’s nutrient acquisition (Artursson et al., 2006). However, the functional mechanisms behind these interactions should be investigated before mixing AMF with specific microorganisms. Importantly, apart from abovementioned synergistic engagements, AMF can also negatively influence certain bacterial populations (i.e., *Actinobacteria*; Nuccio et al., 2013). Anticipating these interactions, it may be interesting to investigate the residing microbiome in the intended farmlands and adjust SynComs to be more compatible with this native microbiome. This topic was not covered in the present meta-analysis as the scope of our research lies with bacteria.

### Bacterial Application Is More Efficient When Directly Introduced Into the Spermosphere

The direct introduction of PSB to the roots by means of seed coating and root dip resulted in the largest increase in plant P-uptake and shoot biomass. By applying microorganisms directly onto the roots, they gain the advantage for early root colonization (prior to exposure to indigenous soil microorganisms) and exploiting the rhizosphere niche. However, in practice, the formulation of a bacterial inoculant has to be cost efficient, and its application easy to handle to be widely adopted in agriculture, which is not the case for the root dip method. Additionally, inoculum formulations should be tailored toward the specific needs of the chosen microorganism(s) in the respective soil to optimize their survival. Although liquid formulations such as soil drench are easy to handle and apply, the lack of a carrier that provides protection and stabilization is a major limitation. The use of solid formulations, in which bacteria are mixed with a carrier, is more robust and bacteria are more likely to persist in the field (Herrmann and Lesueur, 2013). Microbial activity occurring in the spermosphere has long-lasting effects on plant health and development, making seed inoculation an attractive PBS-formulation method from an agricultural perspective (Berlanga-Clavero et al., 2020). Prior research confirms that PSB inoculation by means of seed coating results in larger increases in shoot biomass (Rubin et al., 2017), indicating the improved assimilation of PSB in the rhizobiome.

### Implementation of High Potential Phosphate Solubilizing Bacteria Requires an Alternation in the European Legislation

In this research, the application of *Burkholderia* spp. had the largest effect on plant P-uptake, while *Enterobacter sp.* had the greatest effect on shoot biomass. The plant growth promoting and root colonizing capacity of *Burkholderia* spp. has been well documented in literature, however, the use of *Burkholderia* spp. is often restricted because of their potential hazardous nature as phytopathogens and opportunistic human pathogens (Compant et al., 2008; Eberl and Vandamme, 2016). The closely related genus *Paraburkholderia* might in this respect provide a valuable alternative for future research.

The majority of the studies focus on the application of *Bacillus* spp., which is an easy-to-use and store genus due to its sporulation. Our meta-analysis indicates that future studies should reach beyond these usual suspects. However, to this end there is a need for an altered legislation on the use of bacterial species in agriculture. According to the European Regulation (EU) 2019/1009 on biostimulants, the list of microorganisms that can currently be used as biostimulants is limited to only four species: *Azotobacter* spp., *Rhizobium* spp., *Azospirillum* spp., and mycorrhizal fungi. At a national level, other microbial species can be recognized as biostimulants once there is scientific evidence that the species ensure agronomic efficiency and do not provide a risk to the environment or to human, animal or plant health. However, the legislation hereon differs amongst the different Member States (Sundh et al., 2021). Some countries have specific authorization systems for microbial plant biostimulants, while others do not (Caradonia et al., 2019). This discrepancy has currently restricted the exploitation of a common market for (microbial) biostimulants in Europe.

### Possible Pitfalls in the Current Phosphate Solubilizing Bacteria Selection Pipeline

The most adopted method for selecting high performance PSB consists of a large-scale *in vitro* screening followed by small scale greenhouse and field trials with the best performing isolates. Through this sequential selection pipeline, the majority of the selected isolates fail to deliver consistently under practical conditions, possibly due to their lack of rhizosphere competence (Chauhan et al., 2015). This strategy might also result in loss of slow growing and/or unculturable bacteria with high rhizosphere competence and high P-solubilizing capacity. In this regard, implementing the rhizosphere competence as a trait in selection and enrichment strategies has shown promising results (De Zutter et al., 2021).

The lack of performance under practical conditions is often not reported, resulting in a certain publication bias as observed in the funnel plots (Supplementary Figures 1, 2). This is also reflected in the P-uptake, height, shoot- and root biomass ratios (Figure 3), where a value of 1 represents no effect. Here, numerous datapoints are situated well above 1, which confirms that positive results are more likely to be reported than negative (no-effect) results.

A paramount concern in the first steps of the *in vitro* selection pipeline is the use of inappropriate P-sources during the isolate screenings. In the included studies in this meta-analysis, preliminary screenings were generally conducted in/on medium supplemented with tri-calcium phosphate. Given the pH dependency (and concomitant effectiveness) of PSB, this might be an important pitfall when tailoring the experiments to field trials. Therefore, the P-source used in both *in vitro* and *in planta* preliminary screenings should be a well-considered choice based on the soil physicochemical properties of the intended farmlands, rather than a choice by force of habit. In this meta-analysis, the effectiveness of PSB proved to be better in alkaline soils compared to acidic soils. Generally, in acidic soils the bio-available P-fraction is inherently higher than in alkaline soils, as the free protons in the acidic environment compete with cations for PO_4_^3–^ binding positions (Barrow, 2017; Zheng et al., 2019). Since this process has similar effects as PSB’s organic acid production, PSB’s added value for P-solubilization is diminished. Upon increased soil pH, P is bound to Ca^2+^ or Mg^2+^, whereas the phosphate solubilizing capacity of PSB by means of organic acid production increases.

As previously described by Bashan et al. (2013), a combination of several metal-P compounds (in tandem or together) might form an ecologically relevant alternative. Bearing in mind the pH-zones in which the research used in this meta-analysis was situated (Supplementary Figures 4, 7), the (combined) use of iron- and aluminum phosphate as P-sources poses a relevant representation of the field conditions. Finally, soil chemical characteristics, and particularly soil pH, are known to influence the residing microbial community composition and diversity. Although some phyla remain largely unaffected by soil pH (e.g., *Proteobacteria*), others are strongly influenced (e.g., *Actinobacteria*, *Acidobacteria*, and *Bacteroidetes*) (Lauber et al., 2008; Rousk et al., 2010).

## Conclusion

Due to its highly reactive nature, phosphorus bio-availability is often limited and plants frequently suffer from P-deficiency. The use of PSB as a complement to phosphate fertilizers, or as an alternative of chemical fertilizers to cope with this P-deficiency is uprising. Despite the controversy on the effectiveness of PSB, this meta-analysis proves the added value of PSB in association with a host plant. Finally, future research and applications should take the soil physicochemical properties of the intended region into account, as the effectiveness of PSB is largely dependent on substrate acidity.

## Data Availability Statement

The original contributions presented in the study are included in the article/Supplementary Material, further inquiries can be directed to the corresponding author.

## Author Contributions

KA, MA, JV, LD, and ND provided the concept of the review article. ND performed the literature research, and data exploration and analyses. KA and ND prepared the manuscript. BB provided the concept and graphics of Figure 1. All authors revised the manuscript.

## Conflict of Interest

The authors declare that the research was conducted in the absence of any commercial or financial relationships that could be construed as a potential conflict of interest.

## Publisher’s Note

All claims expressed in this article are solely those of the authors and do not necessarily represent those of their affiliated organizations, or those of the publisher, the editors and the reviewers. Any product that may be evaluated in this article, or claim that may be made by its manufacturer, is not guaranteed or endorsed by the publisher.
