# Supplementary material for: Uncovering New Insights and Misconceptions on the Effectiveness of Phosphate Solubilizing Rhizobacteria in Plants: A Meta-Analysis
Source: Front Plant Sci. 2022 Mar 2;13:858804. doi: 10.3389/fpls.2022.858804 (PMC8924522; doi:10.3389/fpls.2022.858804)
Supplement: Supplementary file 1 [file Data_Sheet_1.docx]

Supplementary Material

# Supplementary Figures

## Supplementary Figure 1

| 1. P-effect | 1. Shoot biomass | 1. Root biomass |
| --- | --- | --- |
| 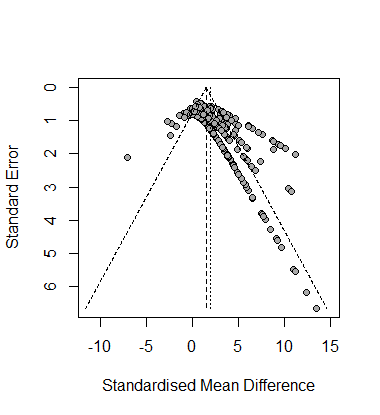 | 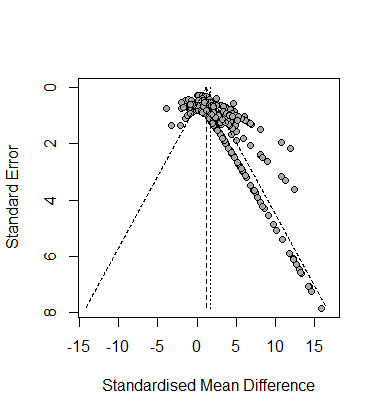 | 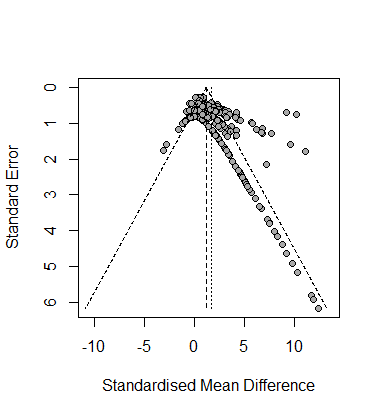 |

**Figure S1** Funnel plots for **(A)** P-uptake, **(B)** shoot biomass and **(C)** root biomass after application of phosphate solubilizing bacteria. Funnel plots represent the standardized mean difference (= effect size) on the x-axis and the concomitant standard error on the y-axis. Each point represents one observation included in the meta-analysis.

## Supplementary Figure 2

| 1. P-effect | 1. Shoot biomass | 1. Root biomass |
| --- | --- | --- |
| 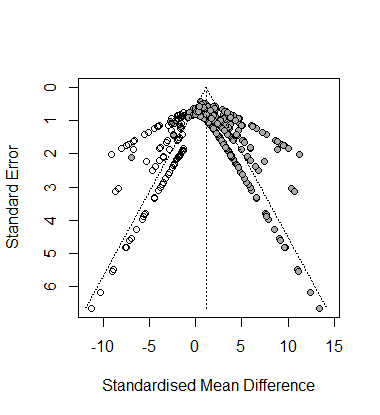 | 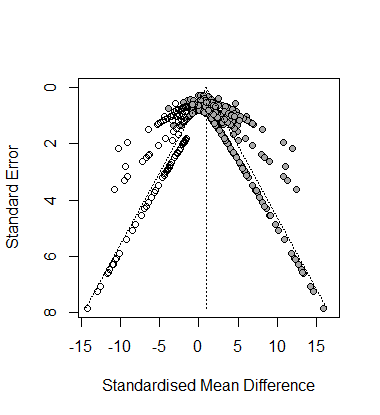 | 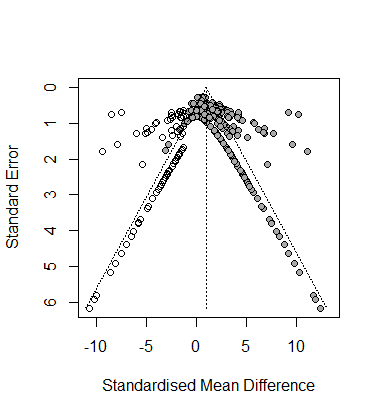 |

**Figure S2** Funnel plots after trimming and filling the data for **(A)** P-uptake, **(B)** shoot biomass and **(C)** root biomass after application of phosphate solubilizing bacteria. Funnel plots represent the standardized mean difference (= effect size) on the x-axis and the concomitant standard error on the y-axis. Each point represents one observation included in the meta-analysis: closed dots represent the original data, open dots represent the filled data.

## Supplementary Figure 3


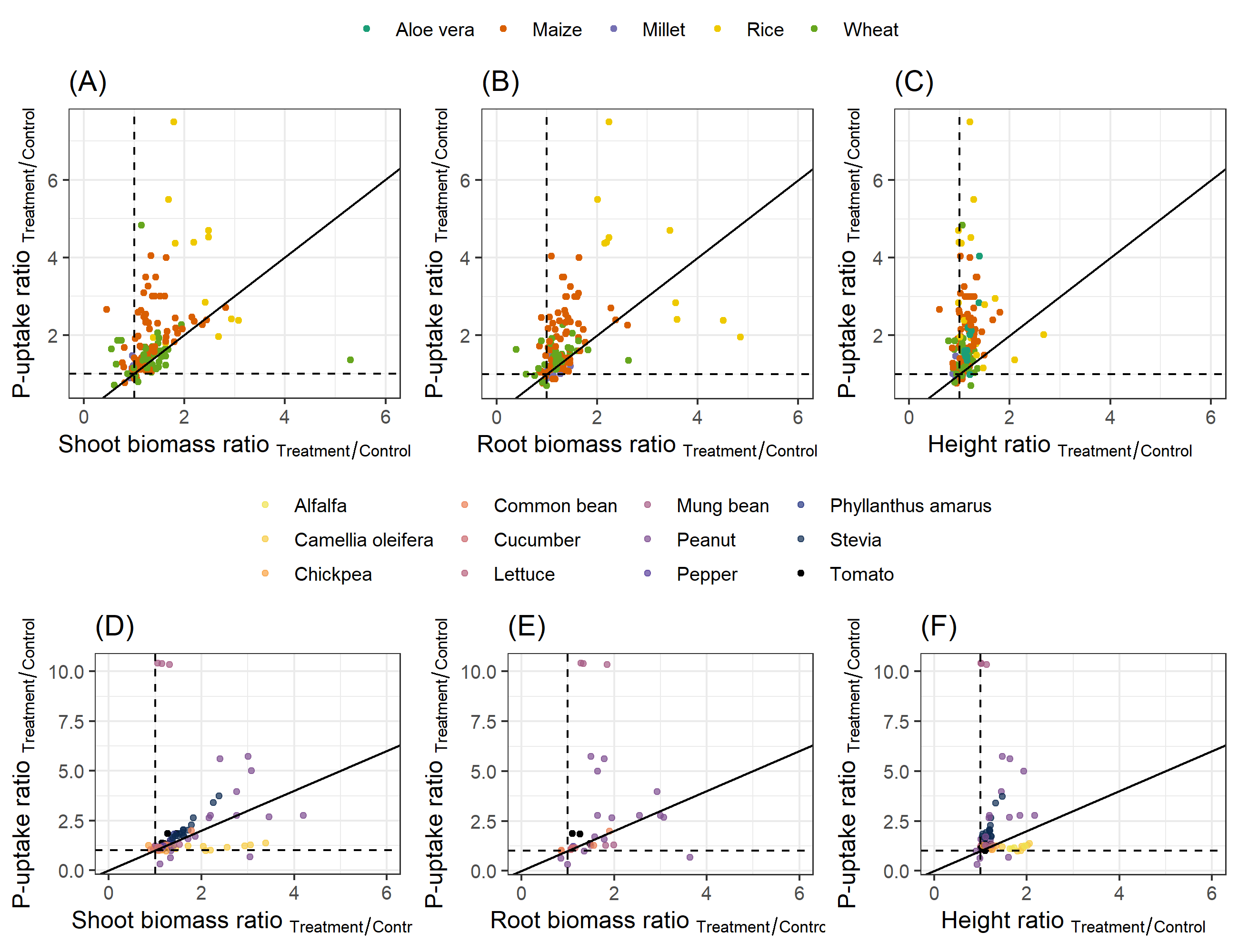


**Figure S3** Relationship between plant P-uptake and **(A, D)** shoot biomass, **(B, E)** root biomass and **(C, F)** length upon bacterial inoculation in monocots **(A-C)** and eudicots **(D-F)**. Values represent the ratio of the means (P-uptake, biomass and length) between the treatment and control.

## Supplementary Figure 4


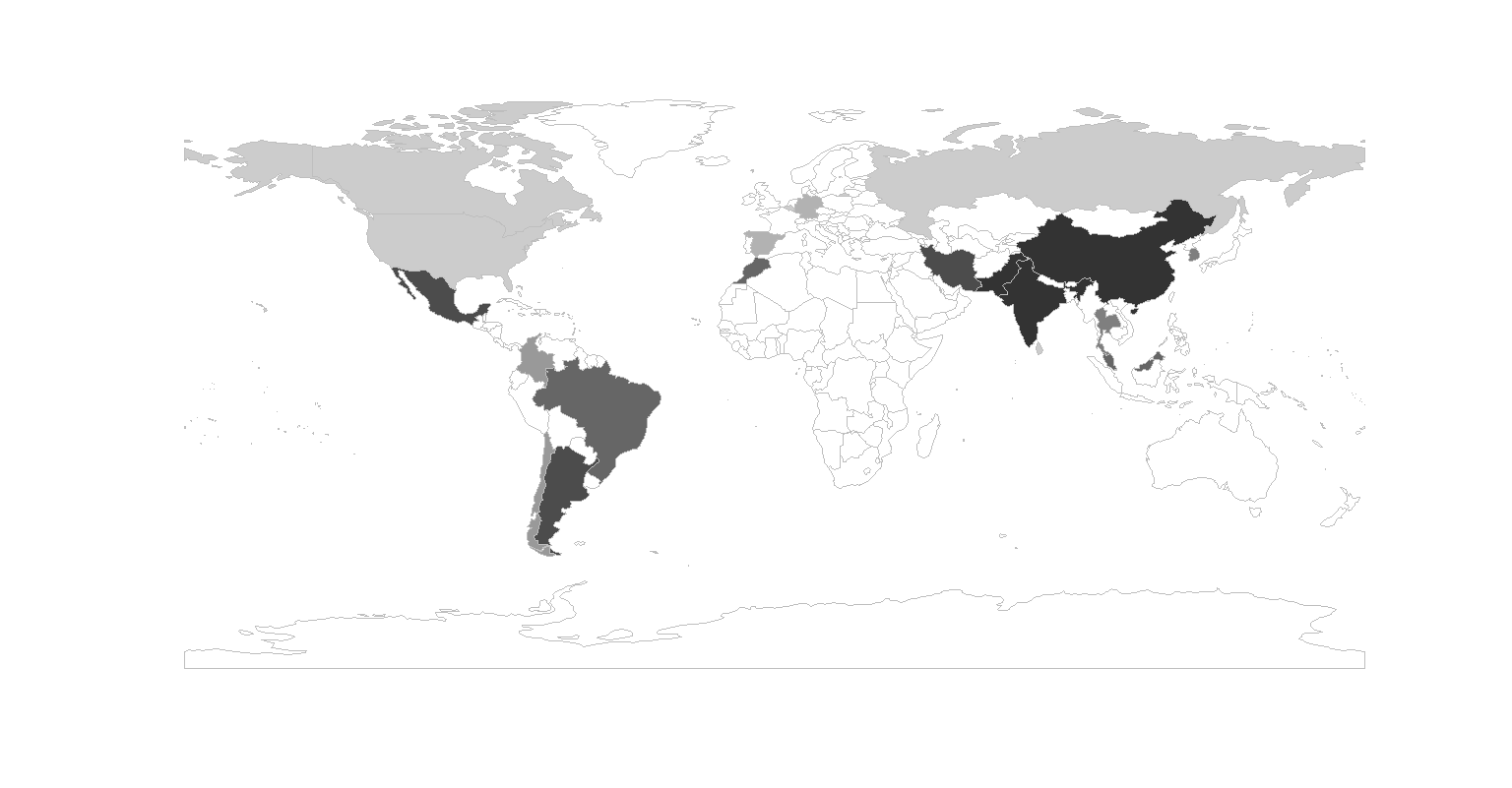


**Figure S4** Origin of the studies used in the meta-analysis. Intensity of the colors represents the amount of studies situated in those countries.

## Supplementary Figure S5


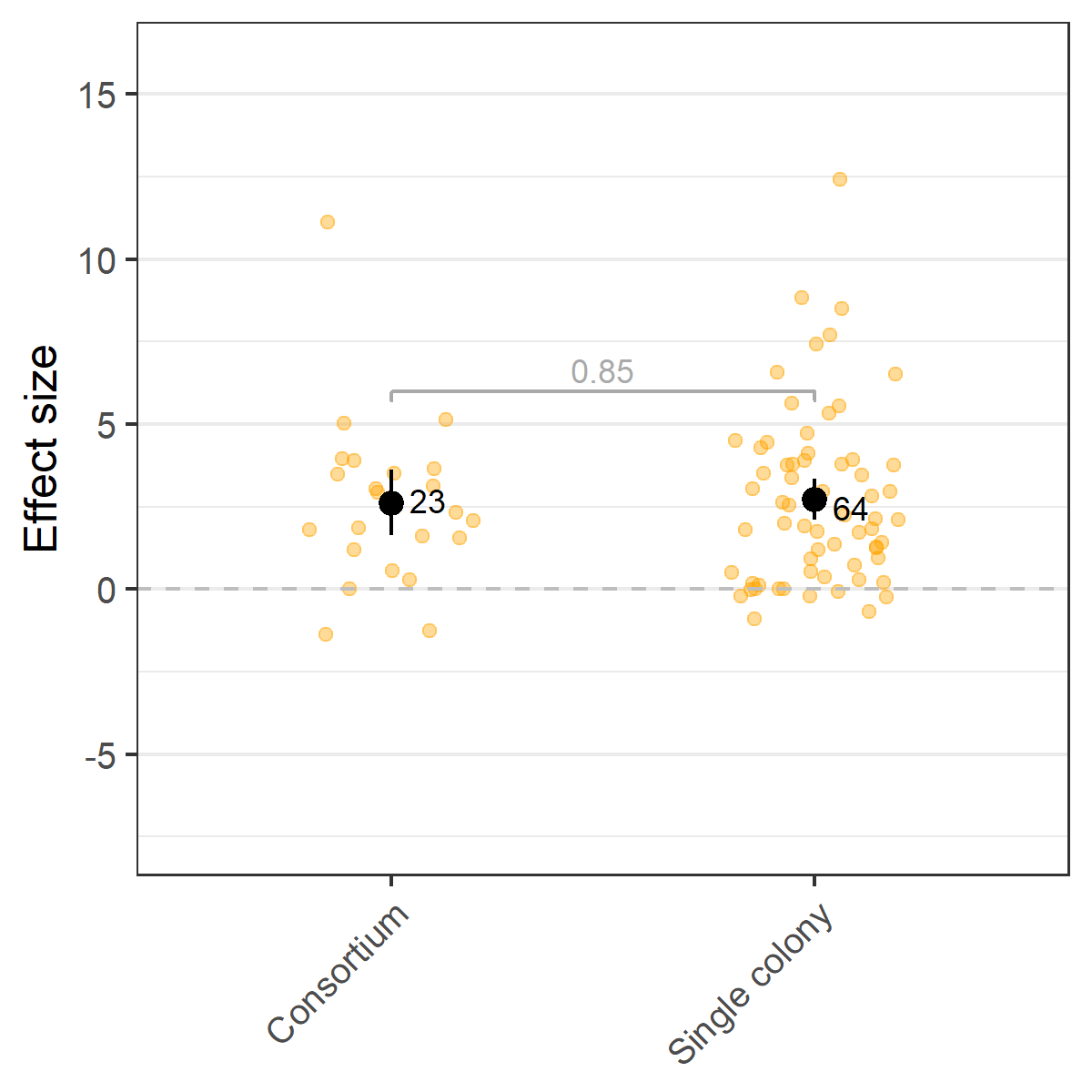


**Figure S5** Effect size on P-uptake upon single and multispecies inoculation performed in the same publication (or a back-to-back paper). Values represent the means ± 95% c.i. Statistical differences were calculated by means of ANOVA.

## Supplementary Figure S6


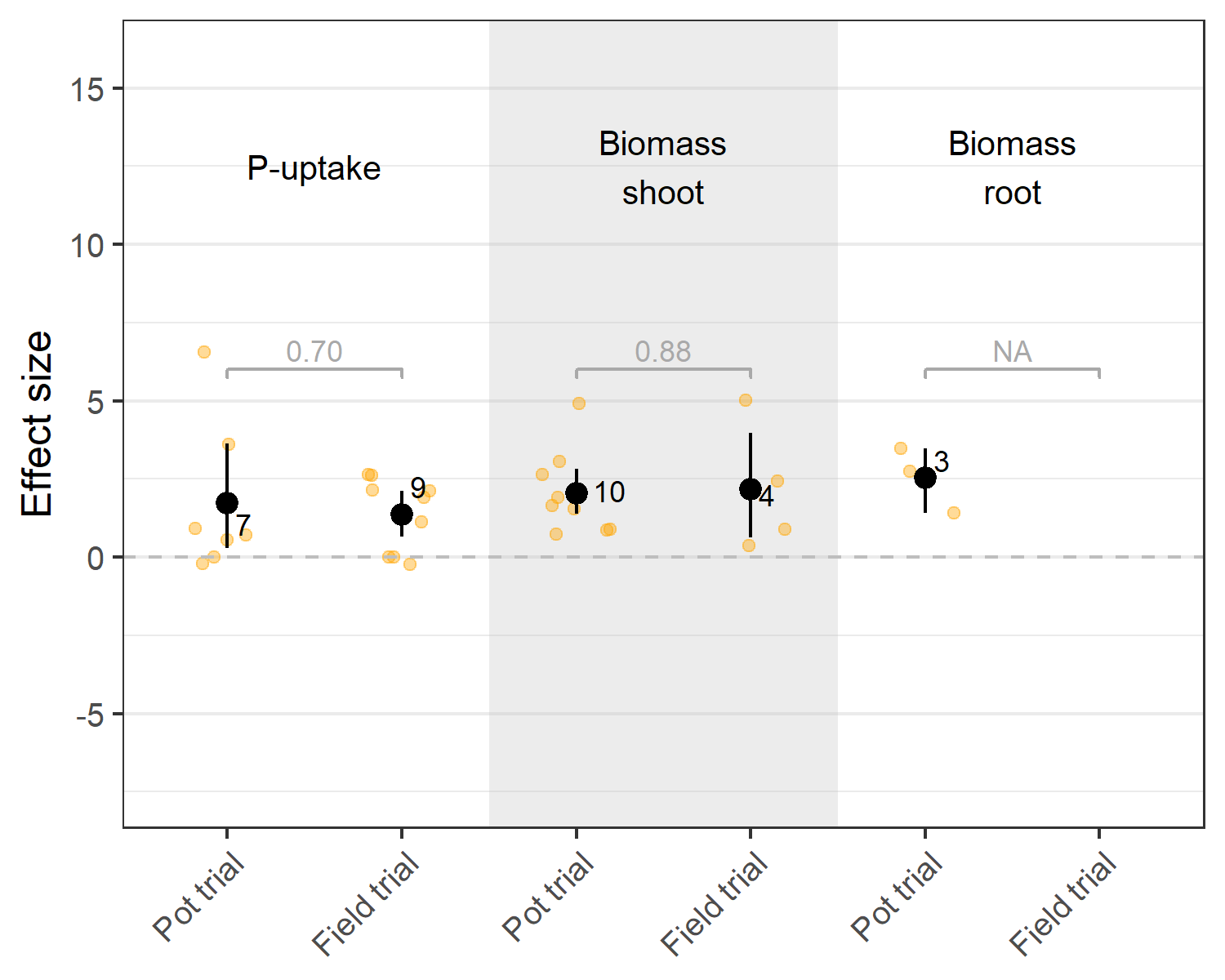


**Figure S6** Effect size on P-uptake upon bacterial inoculation applied in pot and field trials performed in the same publication (or a back-to-back paper). Values represent the means ± 95% c.i. Statistical differences were calculated by means of ANOVA and post-hoc Dunnett T3 tests.

## Supplementary Figure S7


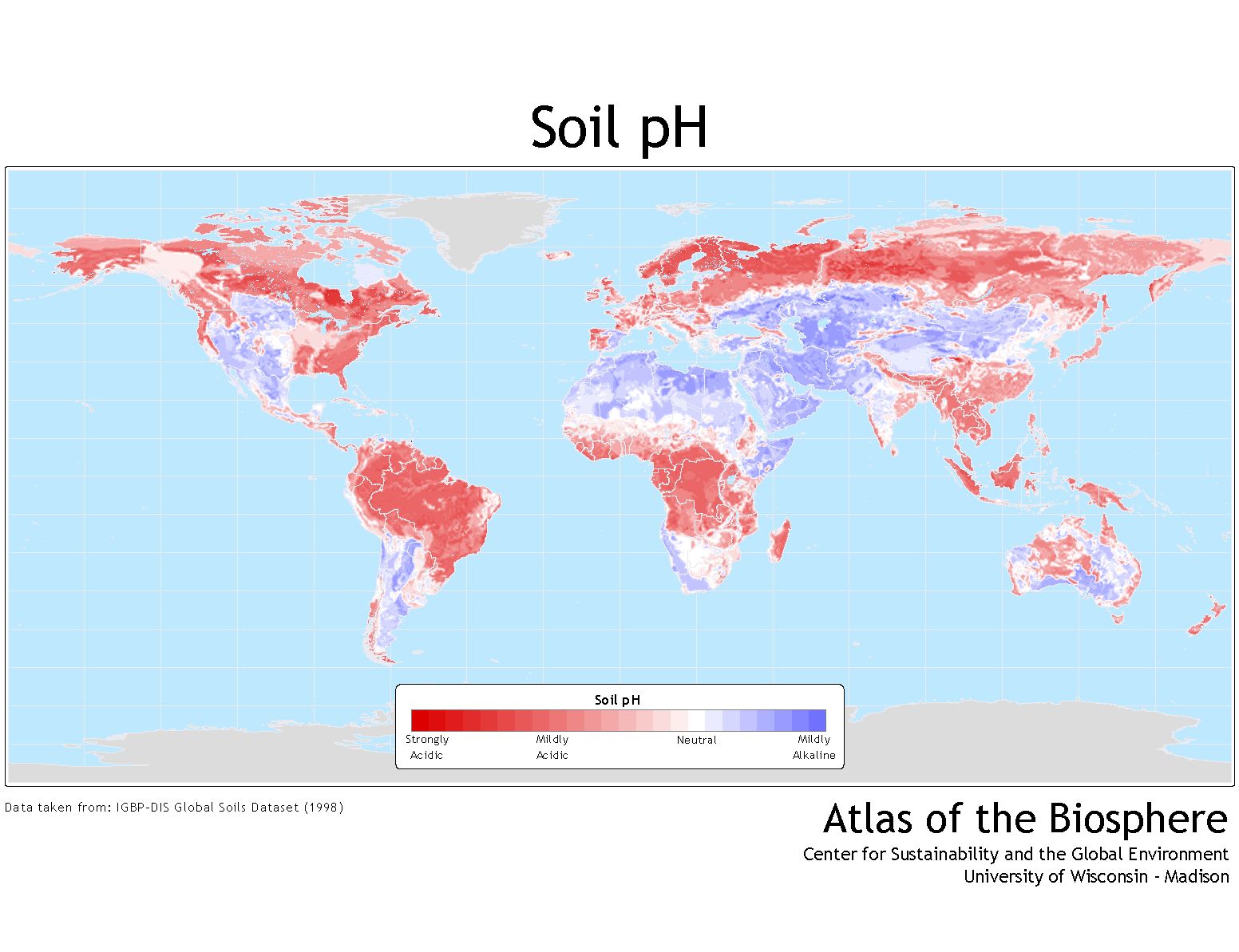


**Figure S7** World map representing global soil pH. Adapted from the Atlas of Biosphere (Center for Sustainability and the Global environment, University of Wisconsin-Madison).

# Supplementary Notes

## Supplementary Note 1

**Note S1** Reference list of the publications used in the meta-analysis.

Abbasi MK, Musa N, Manzoor M. 2015. Mineralization of soluble P fertilizers and insoluble rock phosphate in response to phosphate-solubilizing bacteria and poultry manure and their effect on the growth and P utilization efficiency of chilli (*Capsicum annuum* L.). *Biogeosciences* 12: 4607-4619. <https://doi.org/10.5194/bg-12-4607-2015>

Anzuay MS, Ciancio MGR, Ludueña LM, Angelini JG, Barros G, Pastor N, Taurian T. 2017. Growth promotion of peanut (Arachis hypogaea L.) and maize (Zea mays L.) plants by single and mixed cultures of efficient phosphate solubilizing bacteria that are tolerant to abiotic stress and pesticides. *Microbiological research* 199: 98-109. <https://doi.org/10.1016/j.micres.2017.03.006>

Anzuay MS, Ludueña LM, Angelini JG, Fabra A, Taurian T. 2015. Beneficial effects of native phosphate solubilizing bacteria on peanut (*Arachis hypogaea* L) growth and phosphorus acquisition. *Symbiosis* 66: 89-97. <https://doi.org/10.1007/s13199-015-0337-z>

Arunakumara KKIU, Walpola BC, Yoon MH. 2015. Bioaugmentation-assisted phytoextraction of Co, Pb and Zn: An assessment with a phosphate-solubilizing bacterium isolated from metal-contaminated mines of Boryeong area in South Korea. *BASE* 19: 143-152.

Bakhshandeh E, Pirdashti H, Lendeh KS. 2017. Phosphate and potassium-solubilizing bacteria effect on the growth of rice. *Ecological Engineering* 103: 164-169. <https://doi.org/10.1016/j.ecoleng.2017.03.008>

Bakhshandeh E, Rahimian H, Pirdashti H, Nematzadeh GA. 2015. Evaluation of phosphate‐solubilizing bacteria on the growth and grain yield of rice (*Oryza sativa L.*) cropped in northern Iran. *Journal of applied microbiology* 119: 1371-1382. <https://doi.org/10.1111/jam.12938>

Bautista‐Cruz A, Antonio‐Revuelta B, del Carmen Martínez Gallegos V, Báez‐Pérez A. 2019. Phosphate‐solubilizing bacteria improve Agave angustifolia Haw. growth under field conditions. *Journal of the Science of Food and Agriculture* 99: 6601-6607. <https://doi.org/10.1002/jsfa.9946>

Benbrik B, Elabed A, El Modafar C, Douira A, Amir S, Filali-Maltouf A, *et al.* 2020. Reusing phosphate sludge enriched by phosphate solubilizing bacteria as biofertilizer: Growth promotion of Zea Mays. *Biocatalysis and Agricultural Biotechnology* 30: 101825. <https://doi.org/10.1016/j.bcab.2020.101825>

Bidondo LF, Bompadre J, Pergola M, Silvani V, Colombo R, Bracamonte F, Godeas A. 2012. Differential interaction between two Glomus intraradices strains and a phosphate solubilizing bacterium in maize rhizosphere. *Pedobiologia* 55: 227-232. <https://doi.org/10.1016/j.pedobi.2012.04.001>

Biswas JK, Banerjee A, Rai M, Naidu R, Biswas B, Vithanage M, *et al*. 2018. Potential application of selected metal resistant phosphate solubilizing bacteria isolated from the gut of earthworm (Metaphire posthuma) in plant growth promotion. *Geoderma* 330: 117-124. <https://doi.org/10.1016/j.geoderma.2018.05.034>

Boroumand N, Behbahani M, Dini G. 2020. Combined effects of phosphate solubilizing bacteria and nanosilica on the growth of land cress plant. *Journal of Soil Science and Plant Nutrition* 20: 232-243. <https://doi.org/10.1007/s42729-019-00126-8>

Castagno LN, García IV, Sannazzaro AI, Bailleres M, Ruiz OA, Mendoza RE, Estrella MJ. 2014. Growth, nutrient uptake and symbiosis with rhizobia and arbuscular mycorrhizal fungi in Lotus tenuis plants fertilized with different phosphate sources and inoculated with the phosphate-solubilizing bacterium *Pantoea eucalypti* M91. *Plant and soil* 385: 357-371. <https://doi.org/10.1007/s11104-014-2237-z>

Chen Q, Liu S. 2019. Identification and characterization of the phosphate-solubilizing bacterium Pantoea sp. S32 in reclamation soil in Shanxi, China. *Frontiers in microbiology* 10: 2171. <https://doi.org/10.3389/fmicb.2019.02171>

Collavino MM, Sansberro PA, Mroginski LA, Aguilar OM. 2010. Comparison of in vitro solubilization activity of diverse phosphate-solubilizing bacteria native to acid soil and their ability to promote Phaseolus vulgaris growth. *Biology and fertility of soils* 46: 727-738. <https://doi.org/10.1007/s00374-010-0480-x>

da Costa EM, de Lima W, Oliveira-Longatti SM, de Souza FM. 2015. Phosphate-solubilising bacteria enhance Oryza sativa growth and nutrient accumulation in an oxisol fertilized with rock phosphate. *Ecological engineering* 83: 380-385. <https://doi.org/10.1016/j.ecoleng.2015.06.045>

De Zutter N, Ameye M, Debode J, De Tender C, Ommeslag S, Verwaeren J, *et al*. 2021. Shifts in the rhizobiome during consecutive in planta enrichment for phosphate‐solubilizing bacteria differentially affect maize P status. *Microbial Biotechnology*. <https://doi.org/10.1111/1751-7915.13824>

El Maaloum S, Elabed A, Alaoui-Talibi ZE, Meddich A, Filali-Maltouf A, Douira A, *et al*. 2020. Effect of arbuscular mycorrhizal fungi and phosphate-solubilizing bacteria consortia associated with phospho-compost on phosphorus solubilization and growth of tomato seedlings (Solanum lycopersicum L.). *Communications in Soil Science and Plant Analysis* 51: 622-634. <https://doi.org/10.1080/00103624.2020.1729376>

Estrada-Bonilla GA, Durrer A, Cardoso EJ. 2021. Use of compost and phosphate-solubilizing bacteria affect sugarcane mineral nutrition, phosphorus availability, and the soil bacterial community. *Applied Soil Ecology* 157: 103760. <https://doi.org/10.1016/j.apsoil.2020.103760>

Gupta M, Bisht S, Singh B, Gulati A, Tewari R. 2011. Enhanced biomass and steviol glycosides in Stevia rebaudiana treated with phosphate-solubilizing bacteria and rock phosphate. *Plant Growth Regulation* 65: 449-457. <https://doi.org/10.1007/s10725-011-9615-9>

Gupta M, Kiran S, Gulati A, Singh B, Tewari R. 2012. Isolation and identification of phosphate solubilizing bacteria able to enhance the growth and aloin-A biosynthesis of Aloe barbadensis Miller. *Microbiological research* 167: 358-363. <https://doi.org/10.1016/j.micres.2012.02.004>

Gurdeep KAUR, Reddy MS. 2015. Effects of phosphate-solubilizing bacteria, rock phosphate and chemical fertilizers on maize-wheat cropping cycle and economics. *Pedosphere* 25: 428-437. <https://doi.org/10.1016/S1002-0160(15)30010-2>

Gusain YS, Kamal R, Mehta CM, Singh US, Sharma AK. 2015. Phosphate solubilizing and indole-3-acetic acid producing bacteria from the soil of Garhwal Himalaya aimed to improve the growth of rice. *Journal of environmental biology* 36: 301.

Hameeda B, Harini G, Rupela OP, Wani SP, Reddy G. 2008. Growth promotion of maize by phosphate-solubilizing bacteria isolated from composts and macrofauna. *Microbiological research* 163: 234-242. <https://doi.org/10.1016/j.micres.2006.05.009>

Han HS, Lee KD. 2006. Effect of co-inoculation with phosphate and potassium solubilizing bacteria on mineral uptake and growth of pepper and cucumber. *Plant soil and Environment* 52: 130.

Harris JN, New PB, Martin PM. 2006. Laboratory tests can predict beneficial effects of phosphate-solubilising bacteria on plants. *Soil Biology and Biochemistry* 38: 1521-1526. <https://doi.org/10.1016/j.soilbio.2005.11.016>

Ibarra-Galeana JA, Castro-Martínez C, Fierro-Coronado RA, Armenta-Bojórquez AD, Maldonado-Mendoza IE. 2017. Characterization of phosphate-solubilizing bacteria exhibiting the potential for growth promotion and phosphorus nutrition improvement in maize (Zea mays L.) in calcareous soils of Sinaloa, Mexico. *Annals of Microbiology* 67: 801-811. <https://doi.org/10.1007/s13213-017-1308-9>

Iqbal S, Khan MY, Asghar HN, Akhtar MJ. 2016. Combined use of phosphate solubilizing bacteria and poultry manure to enhance the growth and yield of mung bean in calcareous soil. *Soil & Environment* 35.

Jiang H, Qi P, Wang T, Chi X, Wang M, Chen M, *et al*. 2019. Role of halotolerant phosphate‐solubilising bacteria on growth promotion of peanut (Arachis hypogaea) under saline soil. *Annals of Applied Biology* 174: 20-30. <https://doi.org/10.1111/aab.12473>

Joe MM, Devaraj S, Benson A, Sa T. 2016. Isolation of phosphate solubilizing endophytic bacteria from Phyllanthus amarus Schum & Thonn: Evaluation of plant growth promotion and antioxidant activity under salt stress. *Journal of applied research on medicinal and aromatic plants* 3: 71-77. <https://doi.org/10.1016/j.jarmap.2016.02.003>

Kaleem Abbasi M, Manzoor M. 2018. Biosolubilization of phosphorus from rock phosphate and other P fertilizers in response to phosphate solubilizing bacteria and poultry manure in a silt loam calcareous soil. *Journal of Plant Nutrition and Soil Science* 181: 345-356. <https://doi.org/10.1002/jpln.201800012>

Kaur G, Reddy MS. 2014. Role of phosphate-solubilizing bacteria in improving the soil fertility and crop productivity in organic farming. *Archives of Agronomy and Soil Science* 60: 549-564. <https://doi.org/10.1080/03650340.2013.817667>

Kim KY, Jordan D, McDonald GA. 1997. Effect of phosphate-solubilizing bacteria and vesicular-arbuscular mycorrhizae on tomato growth and soil microbial activity. *Biology and fertility of soils* 26: 79-87. <https://doi.org/10.1007/s003740050347>

Kudoyarova GR, Vysotskaya LB, Arkhipova TN, Kuzmina LY, Galimsyanova NF, Sidorova LV, *et al*. 2017. Effect of auxin producing and phosphate solubilizing bacteria on mobility of soil phosphorus, growth rate, and P acquisition by wheat plants. *Acta physiologiae plantarum* 39: 1-8. <https://doi.org/10.1007/s11738-017-2556-9>

Li H, Li Z, Qu J, Tian H, Yang X. 2018. Combined effects of phosphate-solubilizing bacterium XMT-5 (Rhizobium sp.) and submerged macrophyte Ceratophyllum demersum on phosphorus release in eutrophic lake sediments. *Environmental Science and Pollution Research* 25: 18990-19000. <https://doi.org/10.1007/s11356-018-2022-2>

Li JF, Zhang SQ, Huo PH, Shi SL, Miao YY. 2013. Effect of phosphate solubilizing rhizobium and nitrogen fixing bacteria on growth of alfalfa seedlings under P and N deficient conditions. *Pakistan Journal of Botany* 45: 1557-1562.

Li Y, Li Q, Guan G, Chen S. 2020. Phosphate solubilizing bacteria stimulate wheat rhizosphere and endosphere biological nitrogen fixation by improving phosphorus content. *PeerJ* 8: e9062. <https://doi.org/10.7717/peerj.9062>

Liu FP, Liu HQ, Zhou HL, Dong ZG, Bai XH, Bai P, Qiao JJ. 2014. Isolation and characterization of phosphate-solubilizing bacteria from betel nut (Areca catechu) and their effects on plant growth and phosphorus mobilization in tropical soils. *Biology and fertility of soils* 50: 927-937. <https://doi.org/10.1007/s00374-014-0913-z>

Liu J, Liu X, Zhang Q, Li S, Sun Y, Lu W, Ma C. 2020. Response of alfalfa growth to arbuscular mycorrhizal fungi and phosphate-solubilizing bacteria under different phosphorus application levels. *AMB Express* 10: 1-13. <https://doi.org/10.1186/s13568-020-01137-w>

Liu M, Liu X, Cheng BS, Ma XL, Lyu XT, Zhao XF, *et al*. 2016. Selection and evaluation of phosphate-solubilizing bacteria from grapevine rhizospheres for use as biofertilizers. *Spanish journal of agricultural research* 14: 26. <http://dx.doi.org/10.5424/sjar/2016144-9714>

Lucero CT, Lorda GS, Anzuay MS, Ludueña LM, Taurian T. 2021. Peanut Endophytic Phosphate Solubilizing Bacteria Increase Growth and P Content of Soybean and Maize Plants. *Current Microbiology* 78: 1961-1972. <https://doi.org/10.1007/s00284-021-02469-x>

Magallon-Servín P, Antoun H, Taktek S, Bashan Y, de-Bashan L. 2020. The maize mycorrhizosphere as a source for isolation of arbuscular mycorrhizae-compatible phosphate rock-solubilizing bacteria. *Plant and Soil*, *451*(1), 169-186. <https://doi.org/10.1007/s11104-019-04226-3>

Magallon-Servin P, Antoun H, Taktek S, de-Bashan LE. 2020. Designing a multi-species inoculant of phosphate rock-solubilizing bacteria compatible with arbuscular mycorrhizae for plant growth promotion in low-P soil amended with PR. *Biology and Fertility of Soils* 56: 521-536. <https://doi.org/10.1007/s00374-020-01452-1>

Mahanta D, Rai RK, Dhar S, Varghese E, Raja A, Purakayastha TJ. 2018. Modification of root properties with phosphate solubilizing bacteria and arbuscular mycorrhiza to reduce rock phosphate application in soybean-wheat cropping system. *Ecological Engineering* 111: 31-43. <https://doi.org/10.1016/j.ecoleng.2017.11.008>

Majid M, Ali M, Shahzad K, Ahmad F, Ikram RM, Ishtiaq M, *et al.* 2020. Mitigation of Osmotic Stress in Cotton for the Improvement in Growth and Yield through Inoculation of Rhizobacteria and Phosphate Solubilizing Bacteria Coated Diammonium Phosphate. *Sustainability* 12: 10456. <https://doi.org/10.3390/su122410456>

Mamta G, Rahi P, Pathania V, Gulati A, Singh B, Bhanwra RK, Tewari R. 2012. Comparative efficiency of phosphate-solubilizing bacteria under greenhouse conditions for promoting growth and aloin-A content of Aloe barbadensis. *Archives of Agronomy and Soil Science* 58: 437-449. <https://doi.org/10.1080/03650340.2010.522574>

Meena KK, Mesapogu S, Kumar M, Yandigeri MS, Singh G, Saxena AK. 2010. Co-inoculation of the endophytic fungus Piriformospora indica with the phosphate-solubilising bacterium Pseudomonas striata affects population dynamics and plant growth in chickpea. *Biology and Fertility of Soils* 46: 169-174. <https://doi.org/10.1007/s00374-009-0421-8>

Misra N, Gupta G, Jha PN. 2012. Assessment of mineral phosphate‐solubilizing properties and molecular characterization of zinc‐tolerant bacteria. *Journal of basic microbiology* 52: 549-558. <https://doi.org/10.1002/jobm.201100257>

Nacoon S, Jogloy S, Riddech N, Mongkolthanaruk W, Ekprasert J, Cooper J, Boonlue S. 2021. Combination of arbuscular mycorrhizal fungi and phosphate solubilizing bacteria on growth and production of Helianthus tuberosus under field condition. *Scientific Reports* 11: 1-10. <https://doi.org/10.1038/s41598-021-86042-3>

Nacoon S, Jogloy S, Riddech N, Mongkolthanaruk W, Kuyper TW, Boonlue S. 2020. Interaction between phosphate solubilizing bacteria and arbuscular mycorrhizal fungi on growth promotion and tuber inulin content of Helianthus tuberosus L. *Scientific reports* 10: 1-10. <https://doi.org/10.1038/s41598-020-61846-x>

Ordoñez YM, Fernandez BR, Lara LS, Rodriguez A, Uribe-Velez D, Sanders IR. 2016. Bacteria with phosphate solubilizing capacity alter mycorrhizal fungal growth both inside and outside the root and in the presence of native microbial communities. *PloS one* 11: e0154438. <https://doi.org/10.1371/journal.pone.0154438>

Panhwar QA, Radziah O, Rahman AZ, Sariah M, Razi IM, Naher UA. 2011. Contribution of phosphate-solubilizing bacteria in phosphorus bioavailability and growth enhancement of aerobic rice. *Spanish journal of agricultural research* 3: 810-820.

Panhwar QA, Radziah O, Zaharah AR, Sariah M, Razi IM. 2011. Role of phosphate solubilizing bacteria on rock phosphate solubility and growth of aerobic rice. *Journal of environmental biology* 32: 607.

Parastesh F, Alikhani HA, Etesami H. 2019. Vermicompost enriched with phosphate–solubilizing bacteria provides plant with enough phosphorus in a sequential cropping under calcareous soil conditions. *Journal of Cleaner Production* 221: 27-37. <https://doi.org/10.1016/j.jclepro.2019.02.234>

Qureshi MA, Ahmad ZA, Akhtar N, Iqbal A, Mujeeb F, Shakir MA. 2012. Role of phosphate solubilizing bacteria (PSB) in enhancing P availability and promoting cotton growth. *J. Anim. Plant Sci* 22: 204-210.

Qureshi MA, Shakir MA, Iqbal A, Akhtar N, Khan A. 2011. Co-inoculation of phosphate solubilizing bacteria and rhizobia for improving growth and yield of mungbean (*Vigna radiata* L.). *JAPS, Journal of Animal and Plant Sciences* 21: 491-497.

Rafique M, Sultan T, Ortas I, Chaudhary HJ. 2017. Enhancement of maize plant growth with inoculation of phosphate-solubilizing bacteria and biochar amendment in soil. *Soil science and plant nutrition* 63: 460-469. <https://doi.org/10.1080/00380768.2017.1373599>

Rahi P, Pathania V, Gulati A, Singh B, Bhanwra RK, Tewari R. 2010. Stimulatory effect of phosphate-solubilizing bacteria on plant growth, stevioside and rebaudioside-A contents of Stevia rebaudiana Bertoni. *Applied Soil Ecology* 46: 222-229. <https://doi.org/10.1016/j.apsoil.2010.08.008>

Ren YX, Zhu XL, Fan DD, Ma P, Liang LH. 2013. Inoculation of phosphate solubilizing bacteria for the improvement of lead accumulation by Brassica juncea. *Environmental technology* 34: 463-469. <https://doi.org/10.1080/09593330.2012.701234>

Renseigné N, Han HS, Jung JS, Lee KD. 2006. Rock phosphate-potassium and rock-solubilising bacteria as alternative, sustainable fertilisers. *Agronomy for sustainable development* 26: 233-240. <https://doi.org/10.1051/agro:2006020>

Rezakhani L, Motesharezadeh B, Tehrani MM, Etesami H, Hosseini HM. 2020. Effect of silicon and phosphate-solubilizing bacteria on improved phosphorus (P) uptake is not specific to insoluble P-fertilized sorghum (Sorghum bicolor L.) plants. *Journal of Plant Growth Regulation* 39: 239-253. <https://doi.org/10.1007/s00344-019-09978-x>

Rezakhani L, Motesharezadeh B, Tehrani MM, Etesami H, Hosseini HM. 2019. Phosphate–solubilizing bacteria and silicon synergistically augment phosphorus (P) uptake by wheat (Triticum aestivum L.) plant fertilized with soluble or insoluble P source. *Ecotoxicology and environmental safety* 173: 504-513. <https://doi.org/10.1016/j.ecoenv.2019.02.060>

Rizvi R, Mahmood I, Tiyagi SA. 2013. Potential role of organic matters and phosphate solubilizing bacteria (PSB) on the growth and productivity of fenugreek*. Journal of Agricultural Science and Technology* 15: 639-647.

Sadiq HM, Jahangir GZ, Nasir IA, Iqtidar M, Iqbal M. 2013. Isolation and characterization of phosphate-solubilizing bacteria from rhizosphere soil. *Biotechnology & Biotechnological Equipment* 27: 4248-4255. <https://doi.org/10.5504/BBEQ.2013.0091>

Safirzadeh S, Chorom M, Enayatizamir N. 2019. Effect of phosphate solubilising bacteria (Enterobacter cloacae) on phosphorus uptake efficiency in sugarcane (Saccharum officinarum L.). *Soil Research* 57: 333-341. <https://doi.org/10.1071/SR18128>

Sarikhani MR, Khoshru B, Greiner R. 2019. Isolation and identification of temperature tolerant phosphate solubilizing bacteria as a potential microbial fertilizer. *World Journal of Microbiology and Biotechnology* 35: 1-10. <https://doi.org/10.1007/s11274-019-2702-1>

Saxena J, Jha A. 2014. Impact of a phosphate solubilizing bacterium and an arbuscular mycorrhizal fungus (*Glomus etunicatum*) on growth, yield and P concentration in wheat plants. *CLEAN–Soil, Air, Water* 42: 1248-1252. <https://doi.org/10.1002/clen.201300492>

Saxena J, Saini A, Kushwaha K, Ariño A. 2016. Synergistic effect of plant growth promoting bacterium Pseudomonas fluorescens and phosphate solubilizing fungus Aspergillus awamori for growth enhancement of chickpea. *Indian Journal of Biochemistry and Biophysics* 53: 135-143.

Saxena J, Saini A, Ravi I, Chandra S, Garg V. 2015. Consortium of phosphate-solubilizing bacteria and fungi for promotion of growth and yield of chickpea (*Cicer arietinum*). *Journal of Crop Improvement* 29: 353-369. <https://doi.org/10.1080/15427528.2015.1027979>

Schoebitz M, Ceballos C, Ciamp L. 2013. Effect of immobilized phosphate solubilizing bacteria on wheat growth and phosphate uptake. *Journal of soil science and plant nutrition* 13: 1-10. <http://dx.doi.org/10.4067/S0718-95162013005000001>

Shahid M, Hameed S, Tariq M, Zafar M, Ali A, Ahmad N. 2015. Characterization of mineral phosphate-solubilizing bacteria for enhanced sunflower growth and yield-attributing traits. *Annals of Microbiology* 65: 1525-1536. <https://doi.org/10.1007/s13213-014-0991-z>

Sharma S, Compant S, Ballhausen MB, Ruppel S, Franken P. 2020. The interaction between Rhizoglomus irregulare and hyphae attached phosphate solubilizing bacteria increases plant biomass of Solanum lycopersicum. *Microbiological Research* 240: 126556. <https://doi.org/10.1016/j.micres.2020.126556>

Sharon JA, Hathwaik LT, Glenn GM, Imam SH, Lee CC. 2016. Isolation of efficient phosphate solubilizing bacteria capable of enhancing tomato plant growth. *Journal of soil science and plant nutrition* 16: 525-536. <http://dx.doi.org/10.4067/S0718-95162016005000043>

Shukla A, Kumar A, Chaturvedi OP, Nagori T, Kumar N, Gupta A. 2018. Efficacy of rhizobial and phosphate-solubilizing bacteria and arbuscular mycorrhizal fungi to ameliorate shade response on six pulse crops. *Agroforestry systems* 92: 499-509. <https://doi.org/10.1007/s10457-017-0070-0>

Silva UC, Orellana SC, Silva DRCD, Freitas-Júnior LF, Fernandes AC, Leite LR, *et al*. 2020. Genomic and phenotypic insights into the potential of rock phosphate solubilizing bacteria to promote millet growth in vivo. *Frontiers in Microbiology* 11: 3238. <https://doi.org/10.3389/fmicb.2020.574550>

Singh O, Gupta M, Mittal V, Kiran S, Nayyar H, Gulati A, Tewari R. 2014. Novel phosphate solubilizing bacteria ‘Pantoea cypripedii PS1’along with Enterobacter aerogenes PS16 and Rhizobium ciceri enhance the growth of chickpea (*Cicer arietinum* L.). *Plant growth regulation* 73: 79-89. <https://doi.org/10.1007/s10725-013-9869-5>

Suleman M, Yasmin S, Rasul M, Yahya M, Atta BM, Mirza MS. 2018. Phosphate solubilizing bacteria with glucose dehydrogenase gene for phosphorus uptake and beneficial effects on wheat. *PloS one* 13: e0204408. <https://doi.org/10.1371/journal.pone.0204408>

Sunita S, Kapoor KK, Goyal S, Sharma PK. 2010. Establishment of lacZ marked strain of phosphate solubilizing bacterium in the rhizosphere and its effect on plant growth in mungbean. *Indian journal of microbiology* 50: 117-121. <https://doi.org/10.1007/s12088-010-0068-z>

Surapat W, Pukahuta C, Rattanachaikunsopon P, Aimi T, Boonlue S. 2013. Characteristics of phosphate solubilization by phosphate-solubilizing bacteria isolated from agricultural chili soil and their efficiency on the growth of chili (Capsicum frutescens L. cv. Hua Rua). *Chiang Mai J Sci* 40: 11-25.

Tahir M, Khalid U, Ijaz M, Shah GM, Naeem MA, Shahid M, *et al*. 2018. Combined application of bio-organic phosphate and phosphorus solubilizing bacteria (Bacillus strain MWT 14) improve the performance of bread wheat with low fertilizer input under an arid climate. *Brazilian journal of microbiology* 49: 15-24. <https://doi.org/10.1016/j.bjm.2017.11.005>

Tang A, Haruna AO, Majid NMA, Jalloh MB. 2020. Potential PGPR Properties of Cellulolytic, Nitrogen-Fixing, Phosphate-Solubilizing Bacteria in Rehabilitated Tropical Forest Soil. *Microorganisms* 8: 442. <https://doi.org/10.3390/microorganisms8030442>

Taurian T, Anzuay MS, Angelini JG, Tonelli ML, Ludueña L, Pena D, *et al*. 2010. Phosphate-solubilizing peanut associated bacteria: screening for plant growth-promoting activities. *Plant and Soil* 329: 421-431. <https://doi.org/10.1007/s11104-009-0168-x>

Taurian T, Anzuay MS, Ludueña LM, Angelini JG, Muñoz V, Valetti L, Fabra A. 2013. Effects of single and co-inoculation with native phosphate solubilising strain Pantoea sp J49 and the symbiotic nitrogen fixing bacterium Bradyrhizobium sp SEMIA 6144 on peanut (*Arachis hypogaea* L.) growth. *Symbiosis* 59: 77-85. <https://doi.org/10.1007/s13199-012-0193-z>

Valetti L, Iriarte L, Fabra A. 2018. Growth promotion of rapeseed (Brassica napus) associated with the inoculation of phosphate solubilizing bacteria. *Applied Soil Ecology* 132: 1-10. <https://doi.org/10.1016/j.apsoil.2018.08.017>

Valverde A, Burgos A, Fiscella T, Rivas R, Velazquez E, Rodríguez-Barrueco C, *et al*. 2007. Differential effects of coinoculations with Pseudomonas jessenii PS06 (a phosphate-solubilizing bacterium) and Mesorhizobium ciceri C-2/2 strains on the growth and seed yield of chickpea under greenhouse and field conditions. In *First International Meeting on Microbial Phosphate Solubilization* (pp. 43-50). Springer, Dordrecht. <https://doi.org/10.1007/978-1-4020-5765-6_5>

Viruel E, Erazzú LE, Martínez Calsina L, Ferrero MA, Lucca ME, Siñeriz F. 2014. Inoculation of maize with phosphate solubilizing bacteria: effect on plant growth and yield. *Journal of soil science and plant nutrition* 14: 819-831. <http://dx.doi.org/10.4067/S0718-95162014005000065>

Wahid F, Fahad S, Danish S, Adnan M, Yue Z, Saud S, *et al.* 2020. Sustainable management with mycorrhizae and phosphate solubilizing bacteria for enhanced phosphorus uptake in calcareous soils. *Agriculture*, *10*(8), 334. <https://doi.org/10.3390/agriculture10080334>

Wahid F, Sharif M, Steinkellner S, Khan MA, Marwat KB, Khan SA. 2016. Inoculation of arbuscular mycorrhizal fungi and phosphate solubilizing bacteria in the presence of rock phosphate improves phosphorus uptake and growth of maize. *Pak. J. Bot*, 48: 739-747.

Walpola BC, Yoon MH. 2013. Phosphate solubilizing bacteria: Assessment of their effect on growth promotion and phosphorous uptake of mung bean (Vigna radiata [L.] R. Wilczek). *Chilean Journal of agricultural research* 73: 275. <http://dx.doi.org/10.4067/S0718-58392013000300010>

Wang F, Shi N, Jiang R, Zhang F, Feng G. 2016. In situ stable isotope probing of phosphate-solubilizing bacteria in the hyphosphere. *Journal of experimental botany* 67: 1689-1701. <https://doi.org/10.1093/jxb/erv561>

Wang T, Liu MQ, Li HX. 2014. Inoculation of phosphate-solubilizing bacteria Bacillus thuringiensis B1 increases available phosphorus and growth of peanut in acidic soil. *Acta Agriculturae Scandinavica, Section B–Soil & Plant Science* 64: 252-259. <https://doi.org/10.1080/09064710.2014.905624>

Wang Z, Chen Z, Fu X. 2019. Integrated Effects of Co-Inoculation with Phosphate-Solubilizing Bacteria and N2-Fixing Bacteria on Microbial Population and Soil Amendment Under C Deficiency. *International journal of environmental research and public health* 16: 2442. <https://doi.org/10.3390/ijerph16132442>

Wang Z, Chen Z, Xu Z, Fu X. 2019. Effects of phosphate-solubilizing bacteria and N2-fixing bacteria on nutrient uptake, plant growth, and bioactive compound accumulation in Cyclocarya paliurus (Batal.) Iljinskaja. *Forests* 10: 772. <https://doi.org/10.3390/f10090772>

Wu F, Li J, Chen Y, Zhang L, Zhang Y, Wang S, *et al*. 2019. Effects of Phosphate Solubilizing Bacteria on the Growth, Photosynthesis, and Nutrient Uptake of Camellia oleifera Abel. *Forests* 10: 348. <https://doi.org/10.3390/f10040348>

Xie J, Yan Z, Wang G, Xue W, Li C, Chen X, Chen D. 2021. A Bacterium Isolated From Soil in a Karst Rocky Desertification Region Has Efficient Phosphate-Solubilizing and Plant Growth-Promoting Ability. *Frontiers in microbiology* 11: 3612. <https://doi.org/10.3389/fmicb.2020.625450>

Xu JC, Huang LM, Chen C, Wang J, Long XX. 2019. Effective lead immobilization by phosphate rock solubilization mediated by phosphate rock amendment and phosphate solubilizing bacteria. *Chemosphere* 237: 124540. <https://doi.org/10.1016/j.chemosphere.2019.124540>

Yanez-Ocampo G, Mora-Herrera ME, Wong-Villarreal A, De La Paz-Osorio DM, De La Portilla-Lopez N, Lugo J, *et al*. 2020. Isolated Phosphate-Solubilizing Soil Bacteria Promotes In vitro Growth of Solanum tuberosum L.  *Polish Journal of Microbiology* 69: 357-365. <https://doi.org/10.33073/pjm-2020-039>

You M, Fang S, MacDonald J, Xu J, Yuan ZC. 2020. Isolation and characterization of Burkholderia cenocepacia CR318, a phosphate solubilizing bacterium promoting corn growth. *Microbiological research* 233: 126395. <https://doi.org/10.1016/j.micres.2019.126395>

Zeng Q, Wu X, Wen X. 2017. Identification and characterization of the rhizosphere phosphate-solubilizing bacterium Pseudomonas frederiksbergensis JW-SD2 and its plant growth-promoting effects on poplar seedlings. *Annals of Microbiology* 67: 219-230. <https://doi.org/10.1007/s13213-016-1220-8>

Zhang J, Guo T, Tao Z, Wang P, Tian H. 2020. Transcriptome profiling of genes involved in nutrient uptake regulated by phosphate-solubilizing bacteria in pepper (Capsicum annuum L.). *Plant Physiology and Biochemistry* 156: 611-626. <https://doi.org/10.1016/j.plaphy.2020.10.003>

Zhang J, Wang P, Fang L, Zhang QA, Yan C, Chen J. 2017. Isolation and characterization of phosphate-solubilizing bacteria from mushroom residues and their effect on tomato plant growth promotion. *Polish journal of microbiology* 66: 57-65.

Zhang L, Fan J, Ding X, He X, Zhang F, Feng G. 2014. Hyphosphere interactions between an arbuscular mycorrhizal fungus and a phosphate solubilizing bacterium promote phytate mineralization in soil. *Soil Biology and Biochemistry* 74: 177-183. <https://doi.org/10.1016/j.soilbio.2014.03.004>

Zhang L, Xu M, Liu Y, Zhang F, Hodge A, Feng G. 2016. Carbon and phosphorus exchange may enable cooperation between an arbuscular mycorrhizal fungus and a phosphate‐solubilizing bacterium. *New Phytologist* 210: 1022-1032. <https://doi.org/10.1111/nph.13838>

Zhang T, Hu F, Ma L. 2019. Phosphate-solubilizing bacteria from safflower rhizosphere and their effect on seedling growth. *Open Life Sciences* 14: 246-254. <https://doi.org/10.1515/biol-2019-0028>

Zolfaghari R, Rezaei K, Fayyaz P, Naghiha R, Namvar Z. 2020. The Effect of Indigenous Phosphate-Solubilizing Bacteria on Quercus Brantii Seedlings Under Water Stress. *Journal of Sustainable Forestry* 1-15. <https://doi.org/10.1080/10549811.2020.1817757>
